# Bedside manufacturing as the next step in personalized medicine: medical progress and legal challenges

**DOI:** 10.1093/jlb/lsae012

**Published:** 2024-07-10

**Authors:** Tämer El Saadany, Claudia Seitz, Corina Bräm, Thomas Szucs

**Affiliations:** Division of Oncology/Hematology, Cantonal Hospital Graubünden, Chur, Switzerland; Faculty of Law, University of Basel, Basel, Switzerland; Chirurgie Lindenpark, Kloten, Switzerland; Institute of Pharmaceutical Medicine (ECPM), University of Basel, Basel, Switzerland

**Keywords:** bedside manufacturing, personalized medicine, regulatory frameworks, legal challenges, advanced therapies, orphan diseases

## Abstract

Bedside manufacturing is having a revival in healthcare, with a promise to revolutionize personalized medicine through on-site drug production. While this concept holds considerable promise, it also encounters a complex web of legal uncertainties. The current regulatory framework in Switzerland and the EU, which includes the Swiss Therapeutic Products Act and the EU directives, regulations, and guidelines, fails to adequately address its distinct challenges. Rising new technologies underscore the urgent need for regulatory reform. These technologies highlight the pressing demand for comprehensive legal frameworks that can reconcile the rapid pace of innovation with the imperatives of patient safety and product efficacy. Legal concerns extend beyond mere compliance; they encapsulate quality assurance, and liability in cases of human error. This study outlines the call for a recalibrated legal landscape that prioritizes patient-centered care while fostering the growth of bedside manufacturing. It is crucial for the legal system to evolve in tandem with these medical advancements, ensuring a secure, efficacious, and equitable integration of bedside manufacturing into healthcare.

## I. INTRODUCTION

For centuries doctors have themselves autonomously prepared drugs for their patients. With industrialization and the rise of organic chemistry, large pharmaceutical companies emerged. Drug discovery and production has split from the clinic and has become a major business. For a long time, blockbuster drugs for the treatment of great numbers of patients were produced with the idea of ‘one size fits all’ in mind. In recent decades, the advances in the knowledge of molecular biology and therefore personalized medicine, especially in genomic medicine, have grown substantially. The treatment of patients is becoming more and more individualized. The *Personalized Medicine Coalition* defines personalized medicine as ‘an evolving field in which physicians use diagnostic tests to determine which medical treatments will work best for each patient or use medical interventions to alter molecular mechanisms that impact health. By combining data from diagnostic tests with an individual’s medical history, circumstances, and values, health care providers can develop targeted treatment and prevention plans with their patients’.^1^

In the 1970s, the biotechnology industry was born due to a discovery made by *Herbert Boyer* and *Stanley Norman Cohen*. The two scientists successfully implanted foreign DNA into bacteria causing it to express new proteins. With the help of this recombinant DNA technology, biologicals could be produced for the first time, eg the oligopeptide hormones *somatostatin* or *insulin*.^2^ Biologicals have since become widely used and indispensable in the daily clinical routine. On reflection, however, it turns out that personalized medicine is not yet fully individualized. Rather, drugs are being developed for groups of patients who share the same characteristics in their disease or their genes. For example, *trastuzumab* is only used in HER2 positive breast cancer patients.^3^ Another example is patients with the HLA-B*5701 allele who are not treated with *abacavir* to avoid hypersensitivity reactions.^4^ However, this principle is not useful for small groups or individual patients who suffer from rare diseases. In Europe a disease is considered ‘rare’ if it occurs in one per 2000 people or less:^5^ Pursuant to Article 3(1) of Regulation 141/2000 the disease is ‘affecting not more than five in 10,000 persons in the Community’.^6^ The same definition applies also in Switzerland according to Article 4(1) litera a^decies^ No 1 of the Federal Act on Medicinal Products and Medical Devices (Therapeutics Products Act, TPA).^7^

In the USA a disease is ‘rare’ if it occurs with a prevalence below 200,000 within the population (Section 2(a)(1) of the Rare Disease Act of 2002.^8^ There are approximately 7000 *orphan* or *rare* diseases.^9^ New research results of 2020 confirm that the number of people worldwide living with a rare disease is estimated at 300 million.^10^ As a consequence, unmet needs for patients with a rare disease remain high. It is likely that approximately 80 per cent of these diseases have a genetic origin.^11^ Most of them have no treatment and it is unlikely that treatments will be developed within a reasonable time.^12^ Rolling out new drugs to market has become a complex, time-consuming, and cost-intensive process. In particular the prices of orphan drugs are high, since these prices may reflect the need to recoup the cost of drug development from a small patient pool.^13^ In addition, the rapid growth in approved rare disease treatments in recent years has created concerns about the pricing of orphan drugs and their cumulative affordability to the health system.^14^

In the past various scandals and disasters have occurred in drug development. In 1928 *Elixir sulfanilamide*, a medication for coughing, killed more than 100 people because of the diethylene glycol it contained.^15^ In the 1950s and 1960s more than 10,000 children were born with *dysmelias* because of thalidomide intake of the mothers during pregnancy.^16^ During a phase I study in 2006 with *TeGenero*, participants had to be treated in the intensive care unit because of severe adverse events.^17^ Such occurrences have contributed to the formation of regulating agencies and the adoption of more and more complex regulatory hurdles. The latter have to be fulfilled to evaluate the benefit–risk ratio of a new drug candidate and its eligibility for treatment of patients. Therefore, it has become difficult for academia and small startups to develop new drugs. Bedside manufacturing, also known as point-of-care manufacturing, however is suitable for producing medication on the spot in a short time at comparatively low cost. This would perfectly fit the need of individual patients without high regulatory obstacles. The historical topic of bedside manufacturing is experiencing a revival.

## II. THE POTENTIAL OF BEDSIDE PRODUCTION

What personalized medicine with bedside production might look like was impressively demonstrated in the following case: A six-year-old girl named Mila was presenting with dysarthria, dysphagia, frequent falls, rapid vision loss, and epileptic seizures. She was diagnosed with Batten disease after genetic panel testing. The panel testing revealed a mutation in one allele of the gene CLN7, also called MFSD8. A whole-genome sequencing was then performed. A prior unknown additional mutation in the other allele of gene CLN7 in intron number six was found, a mutation that therefore was impossible to find in the panel testing. Further RNA sequencing revealed a misssplicing in intron 6, leading to a premature termination of the translation.^18^ The autosomal recessive Batten syndrome is also called neuronal ceroid lipofuscinoses. It is a progressive neuropediatric disorder belonging to the group of lysosomal storage diseases. The disease occurs in one out of 100,000 live births. Incorrectly produced autofluorescent material is accumulated in the lysosomes leading to impaired glial reactivity and loss of neurons. Patients die prematurely and suffer from cognitive and motor problems. Also, seizures and visual disturbances, often followed by blindness, depending on the kind of mutation, may occur. In 2017 cerliponase alfa, a therapy for Batten disease has been approved by the FDA. However, this enzyme replacement therapy only works for a gene defect situated in gene CLN2.^19^

Mila’s mutation was located in CLN7 so the idea to develop an antisense oligonucleotide emerged. The already approved drug nusinersen, an antisense nucleotide, changes the misssplicing pattern in spinal muscular atrophy. This drug became the role model for Mila’s treatment. Different antisense nucleotides were developed and tested on Mila’s fibroblasts. The most efficient one, a 22 nucleotide antisense oligonucleotide, was selected and called milasen. In fibroblasts treated with milasen the normal protein amount triplicated and an improvement in the histological picture could be observed. Toxicity of the oligonucleotide was then tested and ruled out in animal trials. Following the scheme of nusinersen, the new drug was given intrathecally to the young patient. The treatment had acceptable side-effect profile and led to a reduction of duration and frequency of epileptic seizures.

A new therapy just for one patient, fitting no one else, had been developed. From the first consultation until the first application of the newly developed drug just one year had passed.^20^ In order to synthesize such an oligonucleotide as described above today, no extensive laboratory is necessary. A team of scientists used a computer, Microsoft Power Point, and a commercially available inkjet printer with the cartridges being loaded with chemical reagents. With just these they successfully synthesized such an oligonucleotide on a microreactor chip.^21^ A benchtop solution for the synthesis of biologicals has been invented by a team working at the MIT. They called their machine InSCyT (Integrated Scalable Cyto-Technology). InSCyT can produce recombinant biologicals automatically in medical grade quality within just three days. The producing organism inside the production module is yeast, pichia pastoris. The attached purification module purifies the substance to an extent of clinical grade. As a next step the protein is sent into a formulation module where the final dosage is prepared. This system was able to produce around 50–75 doses of human growth hormone within 12 hours. In addition, IFN-α (interferon-α) and G-CSF (Granulocyte-Colony Stimulating Factor) have been successfully produced with InSCyT.^22^ Small-scale production can be cheaper than using the original drug by established pharmaceutical companies. This has been shown in a study; recombinant human acid alpha-glucosidase (rhGAA), which is used for the therapy of pompe disease, was produced at small scale. The result was 71 per cent cheaper than the commercially Myozyme™.^23^ Another example of successful bedside production involved a 68-year-old diabetic patient suffering from a severe multidrug-resistant *Acinetobacter baumannii* infection. After identifying several bacteriophages with lytic activity against *A. baumannii*, these were administered to the patient, leading to significant clinical improvement and complete clearance of the infection.^24^ This technology is now being advanced further using CRISPR to enhance the effectiveness of phages, presenting an opportunity to combat the rising threat of antibiotic-resistant infections.^25^

For the evaluation of treatments double-blind randomized controlled trials are the ‘gold standard’. This is often impossible to be applied in studies for rare diseases. There are too few patients and the patients are scattered across national borders around the globe which makes collaboration challenging. In addition, it is more difficult to choose the right endpoints and biomarkers, especially in diseases with no prior treatment. Possible solutions are alternative control groups. An example is baseline controls, in which the patients are compared with what would have been the course of the disease based on historical experience. Another example is the cross-study control where patients are compared with a control group from historical randomized controlled trials. Coordinating multicentric studies around the globe is getting easier with electronic medical records. However, difficulties arise when developing new drugs and planning clinical trials for a few or even single patients. In this case, it would seem necessary to break away from the traditional phase I–III studies. Other concepts like N-of-1 clinical studies, as used for Mila’s treatment, and regulatory flexibility are crucial.^26^

## III. LEGAL CHALLENGES OF BEDSIDE PRODUCTION

### III.A. Bedside Production as a New Type of Drug Manufacturing

Bedside manufacturing as the practice of producing medical products on-site or at the point of care has gained prominence due to its potential to address specific patient needs and overcome supply chain challenges. However, this decentralized manufacturing approach introduces regulatory complexities to ensure the safety, quality, and efficacy of the manufactured products. In general, the regulatory requirements surrounding bedside manufacturing cover legal frameworks, requirements, and monitoring strategies employed by regulatory authorities. Understanding the regulatory landscape is vital for healthcare professionals and manufacturers involved in bedside manufacturing to navigate the compliance process and ensure the highest standards of patient care.

As a general principle in various jurisdictions, all drugs and therapies must be authorized by the competent authorities before they can be marketed and made available to patients. The preventive control of medicinal products and drugs pursues the goal of ensuring that only safe and effective medicinal products reach the market.^27^ In principle the market authorization refers to the drug that is tested in the approval process. The manufacturer or sponsor follows a series of studies—preclinical studies (eg through animal studies) and clinical trials in humans (phase I–III)—to ensure the efficacy of the drug, that is, the medication is safe and the side effects are assessed and that the drug will provide the specific health benefit.^28^ While absolute safety of the drug is not possible and therefore cannot be required, the relative safety is examined in the form of a benefit–risk assessment when testing the effectiveness and safety of the drug.

Legal issues related to advances in bedside manufacturing, however, arise in various regulatory frameworks for drug approval, as these frameworks do not specifically address this new type of drug manufacturing: The current design of the national and European drug approval procedures does not provide for the manufacture of drugs at the bedside and therefore does not contain a specific legal framework for bedside manufacturing. The legal requirements for market authorizations of orphan drugs and the development and approval of a drug for only a few patients do not fit bedside manufacturing either. The regulatory hurdles are therefore currently one of the greatest obstacles to bedside manufacturing and their use for the benefit of patients. Drug approval processes have traditionally been tailored to drugs for an indefinite number of patients. In the case of orphan drugs, the number of patients is usually very small. But even if there are only a few patients for rare diseases, the approval procedure does not refer to an individual patient, but to the respective patient group suffering from the orphan disease.

In the context of bedside manufacturing regulatory agencies play a pivotal role in overseeing the manufacturing and distribution of medical products, including those produced through bedside manufacturing. Authorities such as the European Medicines Agency (EMA) in the EU establish guidelines and regulations that govern the production, labeling, distribution, and use of medical products.^29^ These regulations may apply to both traditional manufacturing facilities and point-of-care manufacturing, depending on the nature and purpose of the products. For bedside manufacturing legal hurdles still exists. These obstacles and challenges, resulting from the current legal frameworks, are examined in the following part from the perspective of Swiss and EU law. Most of the basic problems, however, can be applied to other legal systems with a comparable drug control.

### III.B. Swiss Law

#### III.B.1. Orphan drug solution

Switzerland has a well-established regulatory framework for medical devices and pharmaceuticals. The Therapeutic Products Act (TPA)^30^ and other ordinances and guidelines provide the legal basis for ensuring patient safety and product quality. The Swiss Federal Agency for Therapeutic Products Swissmedic is responsible for overseeing the safety, quality, and efficacy of medical products (Articles 68 et seq. TPA).^31^ As a general principle, ‘only high quality, safe, and effective therapeutic products are placed on the market’ according to Article 1(1) TPA. As a consequence ready-to-use medicinal products may according to Article 9(1) TPA be placed on the market only if authorized by Swissmedic.

As outlined above, bedside manufacturing helps—not exclusively, but primarily—patients suffering from rare diseases. Pursuant to Article 4(1) lit. a^decies^ no. 1 and 2 TPA defines ‘important medicinal products intended to treat rare diseases (orphan drugs)’ as ‘medicinal products for human use for which it has been proven that (1) they are indicated for the diagnosis, prevention, or treatment of a life-threatening or chronically debilitating disease affecting no more than five in 10,000 people in Switzerland when the application was submitted, or (2) they or their active substances are granted the status of important medicinal products intended to treat rare diseases by another country with an equivalent system of medicinal product control within the meaning of Article 13 TPA’.

In general, medicinal products with new active substances (NASs) are authorized in an ordinary procedure in accordance with Articles 10 and 11 TPA. The application for a marketing authorization for an NAS is regulated in Article 11 TPA. An NAS is understood to be a chemical, herbal, biological, biotechnological, or radiopharmaceutical active substance that to date has not been included in any medicinal product that is or was authorized by Swissmedic in an ordinary procedure in accordance with Article 11 TPA (Article 4 para. 1 let. h TPA).^32^ As part of the regular approval process, the quality, safety, and efficacy of the NAS must be assessed on the basis of the submitted data dossier (preclinical and clinical studies). This procedure is therefore time-consuming and costly.^33^ For example, once a pharmaceutical company has filed an application for marketing authorization, it takes an average of almost 650 days for the quality, safety, and efficacy of the drug to be tested by Swissmedic before it is available for a patient.

Because the number of patients with orphan diseases is very small but the costs for a marketing authorization are in general the same the development of innovative and effective treatments of orphan diseases needs to be commercially viable. Since the development and registration of an NAS for an orphan disease is a long and costly venture for a small patient group incentives need to be geared toward improving the return on a particular investment relative to the investment costs, in particular for areas where patients´ needs are still unmet. An incentive is an authorization for medicinal products for rare diseases in a simplified manner: Article 14 TPA provides for a simplified authorization procedure for important medicinal products for rare diseases. For instance, Swissmedic may accept published results instead of complete study reports for orphan drugs.

The implementing provisions have been incorporated into the Ordinance of the Swiss Agency for Therapeutic Products on the Simplified Licensing of Therapeutic Products and the Licensing of Therapeutic Products by the Notification Procedure (TPLO).^34^ A distinction is made in the Ordinance between recognition of the status as an important medicinal product for rare diseases (Article 4 to 7 TPLO) and the authorization of a medicinal product that has been granted Orphan Drug Status by Swissmedic (Article 24 to 26 TPLO). In principle, however, the authorization procedure for an orphan drug corresponds a lot to the procedure provided for the respective category of different medicinal products. Quality, safety, and efficacy must be assured. In contrast to a non-rare disease, the collection of preclinical and clinical data is much more difficult due to the rarity of the disease. This is a fact that is taken into account in the assessment by Swissmedic.^35^ A list of medicinal products with orphan drug status is published by Swissmedic.^36^ No special acceleration of the procedure is foreseen, even if the dossiers are prioritized as far as possible.

#### III.B.2. Magistral solution

As mentioned above, generally every drug or medicinal product must be authorized before its usage pursuant to Article 9(1) TPA. However, so-called ‘formula medical products’ are excluded from the authorization (Article 9(2) TPA). According to Article 9(a) TPA medicinal products prepared according to a doctor’s prescription by a public pharmacy or a hospital pharmacy, or under mandate to the latter by another establishment holding a manufacturing license, and for a given person or group of persons are exempted from authorization. These magistral formulas that are as medicinal products exempted from authorization are prepared in a public pharmacy or a hospital pharmacy according to a medical prescription for a particular person or a particular group of people. Magistral formulas are mainly produced in small quantities for their customers.

In addition, there is also a category of non-standardized medicinal products in accordance with Article 9(2) lit. e TPA. Due to their origin and biological variability, they are not equally standardizable as conventional medications. Non-standardized medicinal products do not correspond to the definitions of ‘formula medicinal products’, although they are also produced on prescription on a patient-specific basis. In contrast to ‘formula medicinal products’ they are usually obtained and/or produced according to a standardized process. Non-standardized medicinal products are usually not produced in pharmacies and their manufacture and placing on the market requires an operating license from Swissmedic. In addition, Swissmedic designates a certain amount of these non-standardized medicinal products with an insufficiently known safety or efficacy profile or with an increased risk due to their composition, dosage, or application. Swissmedic subjects these products to an authorization requirement for the production or manufacturing process (list in Annex 3, Article. 42a VAZV).^37^ This list includes, for example, Platelet Rich Plasma therapy or organ cell extracts of animal origin or fecal microbiome preparations.

In view of the risk potential of such innovative therapeutic approaches, in this manner Swissmedic aims to ensure sufficient quality, safety, and efficacy of such more patient-specific preparations. Medication produced ‘bedside’ eg similar to milasen will most probably—considering the risk profile and relatively complex technical requirements—not benefit from authorization exemption as a ‘formula medication’. (The principle of formula manufacturing is akin to bedside manufacturing, characterized by the term ‘formula’, which signifies *n* = 1, for a specific patient.) It seems more likely that such medication either goes through an approval process with certain simplifications in data to be submitted and possible prioritization or they fall in the category of non-standardized medications (Article 9(2) lit. e TPA) with the subcategory that requires authorization for the production or manufacturing process because of the increased risk profile of such innovative therapies.

#### III.B.3. Freedom of therapy solution

Within the framework of the freedom of therapy, a doctor can suggest an experimental therapy to the patient. As such bedside manufacturing could fall under the categories of ‘attempt at healing’ (ärztlicher Heilversuch) or ‘experimental therapy’. For both categories, however, it is difficult to define what should be covered by them. In particular for experimental therapies it is often not possible to demonstrate that it is an experimental and not a standard therapy. In addition, there is also no commonly shared understanding of what is covered by these categories.^38^ At the federal level there is no specific comprehensive regulation of the ‘attempt at healing’. However, it could be argued that under both categories the use of such medicinal products is only to be considered on an individual basis and in severe cases. However, this individual therapeutic attempt does not replace the market authorization procedure in general as outlined above. A very careful assessment of the benefit/risk ratio must be performed prior to initiating a therapy as an individual healing attempt.

Since patients should preferably be treated within clinical trials the therapeutic attempt constitutes an individual therapy and as such an exemption to the general obligations of authorization. Before such a proposal for an individual therapeutic attempt the doctor must compare the standard therapy and the experimental therapy and is obliged to conduct and document a risk–benefit assessment. This leaves uncertainties for the doctors in cases of bedside manufacturing, in particular in cases where no standard therapy exists.

### III.C. EU Law

#### III.C.1. Pharmaceutical Strategy for Europe 2020

In 2020 the European Commission has already published its ‘Pharmaceutical Strategy for Europe’ to increase access to innovative and safe medicine, including steps to adapt regulation to advanced therapies.^39^ Adopted on November 25, 2020, the Pharmaceutical Strategy for Europe aims at creating a future proof regulatory framework and at supporting the industry in promoting research and technologies that reach patients to fulfill their therapeutic needs while addressing market failures.

Within the subsection of the strategy entitled ‘Enabling innovation and digital transformation’, the first example of innovation given is advanced therapies. In particular, the European Commission states that cell and gene therapies require a regulatory response, noting the volume of such treatments in development. In relation to manufacture, the European Commission suggests that there is a shift from therapeutic products being produced in a traditional factory setting toward a ‘bedside manufacturing’ process. The strategy notes that these new treatment modalities are able to improve patient outcomes and also speed up production time. It also outlines, however, that bedside manufacturing can ‘create new challenges in terms of appropriate quality, inspection, and oversight’. The strategy, however, does not suggest a proposal for a regulation of bedside or point-of-care manufacturing.

#### III.C.2. EU Pharmaceutical Package 2023

EU has established comprehensive regulations to ensure the safety, quality, and efficacy of medical devices and pharmaceuticals. On April 26, 2023, the European Commission put forward a ‘pharmaceutical package’ to revise the pharmaceutical legislation of the EU and make medicines more available, accessible, and affordable while supporting the competitiveness and attractiveness of the EU pharmaceutical industry, with higher environmental standards. The package includes proposals for a new directive and a new regulation, which would replace the existing pharmaceutical legislation, including the legislation on medicines for children and for rare diseases.^40^

#### III.C.3. Missing regulatory scheme for bedside manufacturing

Regulatory authorities in the EU Member States as well as the EMA have widely considered the bedside manufacturing and the production of therapies in hospitals as an exceptional case, and have therefore overseen it by means of regulatory exemptions. In the EU, the EMA has used the so-called hospital exemption, a regulatory scheme that enables clinicians to produce therapies at very small scale to treat specific patients. However, these schemes are now proving limited in scope, as bedside manufacturing is expected to be practiced more widely and frequently, making regulators feel the need for more specific frameworks.

For advanced therapy medicinal products (ATMPs) the European Commission set up a regulatory scheme. ATMPs comprise gene therapies, tissue engineered products, and somatic cell therapies. They have the potential to reshape the treatment of a wide range of conditions, particularly in disease areas where conventional approaches are inadequate. ATMP treatments may help in cases of severe, untreatable, or chronic diseases, such as cancers, cardiovascular diseases, musculoskeletal conditions, and immune system disorders.

In the EU, Regulation (EC) No 1394/2007 has been in force since 2008, providing a common regulatory framework for ATMPs.^41^ The Regulation 1394/2007 provides a centralized authorization and an automatic access to a central route for an EU authorization for ATMPs, according to which ATMPs are authorized in all EU Member States. Other promoting innovative technologies, in particular bedside manufacturing and closed systems, are not sufficiently covered in the Regulation 1394/2007. This would require more flexibility.^42^ This Regulation, however, does not provide a specific regulation for bedside manufacturing or point-of-care treatments.

#### III.C.4. EMA Regulatory Science to 2025

The EMA has issued a Scientific Committees Regulatory Science Strategy in 2018, the ‘EMA Regulatory Science to 2025’.^43^ The objective of this strategy is to respond ‘to the needs of the 21^st^ century Patient’ and address ‘challenges and opportunities across the European Regulatory Framework and leveraging research and innovation in regulatory science’.^44^ The EMA defines ‘regulatory science’ as ‘range of scientific disciplines that are applied to the quality, safety, and efficacy assessment of medicinal products and that inform regulatory decision-making throughout the lifecycle of a medicine’.^45^ The EMA lists the challenges for the regulations of ATMPs, such as a consistent manufacturing of a product across development, clinical use and commercialization, and other issues in relation to cell-based products.

In this context the EMA also mentions the objective to facility the implementation of novel manufacturing technologies in the context of point-of-care and bedside manufacturing. The EMA emphasizes that ‘the pharmaceutical industry is implementing a suite of novel manufacturing technologies to improve cost efficiency and customization’ with the opportunity ‘to tailor production to specific medical needs, particular for innovative products’.^46^ According to the EMA these new approaches ‘range from continuous manufacturing, with a full centralized process, to various models of distributed, local manufacturing and point-of-care/bed-side manufacturing’.^47^ In this context the EMA suggests various underlying actions, such as to ‘address regulatory challenges in point-of-care manufacturing, eg responsibility for manufacturing process, concept of batch control, role of the Qualified Person’.^48^

## IV. RESULTS AND GUIDELINES FOR BEDSIDE MANUFACTURING

### IV.A. Results under Swiss and EU Law

Bedside manufacturing is currently not sufficiently regulated under legal frameworks. As shown above, existing legal instruments under the TPA or the EU regulations do not address bedside manufacturing. This results in legal uncertainty, leaving the risk for bedside manufacturing with the doctor. Within the framework of the freedom of therapy, a doctor can carry out an experimental therapy. However, this is not an adequate solution. First, the benefit–risk assessment is required, which is particularly difficult to carry out when there are no standard therapies that can be used for this assessment. Second, there is legal uncertainty related to bedside manufacturing, which may leave the doctor in an uncomfortable position. Third, bedside manufacturing may raise concerns about maintaining consistent product quality of the drug. In this case it is important to adhere to the Good Manufacturing Practices (GMP) to ensure that the treatment meets the required standards. Fourth, bedside manufacturing might also lead to specific intellectual property considerations as this treatment might involve onsite adaption of patented technologies. The magistral approach, for example, may sidestep intellectual property rules because several patent legislations, eg in the USA, contain an exemption for personal use. In any case, the doctor–patient confidentiality may keep magistral production out of the public domain, making it difficult for patent holders to discover a patent infringement. Precisely by definition, these are not established standard therapies, but rather attempts at healing, the scientific basis of which is usually much more difficult to establish. And fifth, the experimental therapy is a case-by-case solution under specific circumstances and it is not indented to regulate a series of individual treatments such as bedside manufacturing.

### IV.B. Proposals for Guidelines for Bedside Manufacturing

Bedside manufacturing, the practice of producing medical products on-site or at the point of care, has gained prominence due to its potential to address specific patient needs and overcome supply chain challenges. The legal analysis above shows the importance of balancing flexibility and innovation with the need for robust regulatory oversight to protect patient safety and public health, to guide doctors and patients, and to provide legal certainty for bedside manufacturing. A comprehensive understanding of the regulatory requirements and compliance guidelines is crucial for healthcare professionals and manufacturers engaged in bedside manufacturing. The existing legal frameworks, however, do not address bedside manufacturing and its complex legal questions to ensure the safety, quality, and efficacy of the manufactured products. Thus, the most important proposal is to set up a regulatory framework for bedside manufacturing that regulates the point-of-care specialties.

In the meantime, there are only a few guidelines for bedside-manufacturing, such as to ensure quality control and compliance to ensure the consistent production of safe and effective medical products, such as to adhere to GMP or similar quality assurance standards. These standards encompass aspects such as facility design, personnel training, documentation practices, quality control testing, and product traceability. Manufacturers engaged in bedside manufacturing must implement robust quality control systems to meet these requirements. In addition, there might be specific reporting obligations from regulatory authorities for manufacturers engaged in bedside manufacturing. Manufacturers are required to report adverse events, product defects, or any other safety concerns to the regulatory authorities. These reports might help in monitoring the safety and performance of medical products, enabling swift actions to mitigate risks and protect patient safety. Finally, the bedside manufacturing should conduct post-market surveillance to monitor the performance, safety, and effectiveness of medical products produced through bedside manufacturing. This involves ongoing evaluation of real-world data, surveillance systems, and post-market studies. By assessing the safety and efficacy of the products in real-world settings, regulatory authorities can identify emerging risks, address safety concerns, and make informed decisions regarding product labeling, warnings, or recalls.

## V. RESULTS AND OUTLOOK

Where might the future lead? With CRISPR-Cas9, a powerful genetic editing tool has become available that is functioning with low costs. It seems safe to assume that bedside production will occur more and more often. The required tools, small-scale drug production systems, are finding their way into the market, eg the award winning BioXp™ 3250 system, which enables the user to perform on-demand overnight DNA assembly and amplification.^49^ Another example is the Kilobaser, an oligonucleotide-synthesis machine. It delivers the desired product within just two hours.^50^ A team of scientists of Synthetic Genomics, Inc in California have even presented a digital-to-biological converter. It converts digital information such as DNA sequences into DNA, RNA, and proteins.

Even more complex macromolecules like virus particles are produced by the system in a fully automated way. A team was able to successfully synthesize among others a DNA-fragment coding for GFP, abatacept, ranibizumab, trastuzumab, a RNA vaccine, and a bacteriophage. For the synthesis of the polypeptides a cell-free lysate was used and thereby the bioreactor was gotten rid of.^51^ Using small-scale drug production systems with small bioreactors a high volumetric productivity is needed. This necessitates the host cells to be selected accordingly. A shift from Chinese hamster ovary cells (CHO), which are widely used for FDA approved biologicals, to yeast may fulfill the criteria. CHO cells are complicated regarding the required media and storage. Additionally, the process until the product is being released is time-consuming, making it hard-pressed for an alternative.^52^ We may be presented with such an organism with the yeast pichia pastoris. It is growing fast to high densities, efficiently secreting recombinant proteins yet only small amounts of its own proteins. Also, it is capable of humanlike posttranslational processing and it offers only a small risk of viral infection. Furthermore, it is a validated host organism for the manufacturing of certain drugs by the EMA and FDA.^53^

Another crucial issue is the cost: will bedside production ever be affordable and cost-effective? According to an estimation, the bedside production per gram protein costs around €2000—a small amount, when compared with up to €500,000, which is currently paid per gram of biopharmaceuticals.^54^ Insurance companies seem to be convinced by the idea of bedside production. In the Netherlands, the three insurance companies CZ, Zilveren Kruis, and Menzis are financing the project ‘proof of principle’. The project of Utrecht University tries to demonstrate the realization and cost efficacy of biosimilar bedside production.^55^ Production of biologicals and the treatment of orphan diseases seem especially suitable for bedside production. This is due to the fact that the means and the knowledge are already available today. Of course, there will be further hurdles to overcome; the hygienical setting in hospital pharmacies and dealing with human error will have to be regulated. For industry standards this is guaranteed today by the GMP.

Bedside manufacturing also bears the potential for a plethora of new business ideas. Modular production units with exchangeable parts which could be suited to individual needs are conceivable. Bedside production is not yet very prevalent. However, there is a big potential for it to profoundly revolutionize the personalized medicine, thus giving the medical science the boost to help many patients who could not be helped before. Nevertheless, as *Huub Schellekens*, professor of pharmaceutical biotechnology at Utrecht University pointed out: ‘There will always be a need for a pharmaceutical industry for large patient populations and for mass production of drugs.’^56^

Against this background bedside manufacturing should be specifically addressed in regulatory frameworks. Regulation plays a critical role in ensuring the quality, safety, and efficacy of medical products manufactured at the bedside. Finding the right balance between flexibility and innovation while maintaining strong regulatory oversight is crucial to fostering the evolution of bedside manufacturing. Healthcare professionals and manufacturers engaged in bedside manufacturing must have a thorough understanding of the regulatory requirements and compliance guidelines to ensure the highest standards of patient care and public health. Further research and collaboration between regulatory authorities and stakeholders as well as robust legal frameworks are needed to address the evolving landscape of bedside manufacturing and promote patient-centered innovation.

## Footnotes

1 Personalized Medicine Coalition (PMC), T.P.M.C. *What Is Personalized Medicine*? 2021 [cited 2021 02.16.]; Available from: http://www.personalizedmedicinecoalition.org/Education/The_Basics.

2 B. Sands & R. Brent, *Overview of Post Cohen-Boyer Methods for Single Segment Cloning and for Multisegment DNA Assembly*, 113(1) Curr Protoc Mol Biol 3 26 1–3 26 20 (2016).

3 S. Maximiano et al., *Trastuzumab in the Treatment of Breast Cancer,* 30(2) BioDrugs 75–86 (2016).

4 S. Mallal et al., *HLA-B*5701 screening for hypersensitivity to abacavir*, 358(6) N Engl J Med 568–79 (2008).

5 C. F. Wright, D. R. Fitz Patrick & H. V. Firth, *Paediatric genomics: diagnosing rare disease in children*, 19(5) Nat Rev Genet 325 (2018).

6 Regulation (EC) No 141/2000 of the European Parliament and of the Council of 16 December 1999 on orphan medicinal products, OJ L 18, 22.01.2000, p. 1–5, https://eur-lex.europa.eu/legal-content/EN/TXT/PDF/?uri=CELEX:32000R0141&from=EN.

7 Federal Act on Medicinal Products and Medical Devices (Therapeutic Products Act, TPA), of 15 December 2000 (SR 812.21), https://www.fedlex.admin.ch/eli/cc/2001/422/en. Between 6000 and 8000 different rare diseases affect an estimated 30 million people in the EU.

8 Rare Diseases Act of 2002, https://www.govinfo.gov/content/pkg/PLAW-107publ280/html/PLAW-107publ280.htm. See also R. C. Griggs et al., *Clinical research for rare disease: opportunities, challenges, and solutions*, 96(1) Mol Genet Metab 20–6 (2009).

9 C. F. Wright, D. R. Fitz Patrick, H. V. Firth, *Paediatric genomics: Diagnosing rare disease in children*, 19(5) Nat Rev Genet 325 (2018); R. C. Griggs, et al., *Clinical research for rare disease: opportunities, challenges, and solutions*, 96(1) Mol Genet Metab 20–6 (2009).

10 S. Nguengang Wakap et al., *Estimating cumulative point prevalence of rare diseases: analysis of the Orphanet database*, 28 European Journal of Human Genetics 165–173 (2020), https://www.nature.com/articles/s41431-019-0508-0.

11 C. F. Wright, D. R. Fitz Patrick, H. V. Firth, *Paediatric genomics: diagnosing rare disease in children*, 19(5) Nat Rev Genet 325 (2018).

12 B. Klimova, et al., *Global View on Rare Diseases: A Mini Review*, 24(29) Curr Med Chem 3153–8 (2017).

13 K. Jayasundara, et al., *Estimating the clinical cost of drug development for orphan versus non-orphan drugs*, 14 Orphanet Journal of Rare Disesases 1–10 (2019).

14 See for an overview of the costs in the US C. Pearson, L. Schapiro, S. Pearson, *The Next Generation of Rare Disease Drug Policy: Ensuring Both Innovation and Affordability,* 2022, https://icer.org/wp-content/uploads/2022/04/ICER-White-Paper_The-Next-Generation-of-Rare-Disease-Drug-Policy_040722.pdf.

15 C. Hodel, R. Bass, *Are newer scientific concepts in regulatory toxicology used timely and appropriately?* 64–65 Spec No. Toxicol Lett 149–55 (1992).

16 E. Newbronner, K. Atkin, *The changing health of Thalidomide survivors as they age: A scoping review*, 11(2) Disabil Health J 184–91 (2018).

17 T. Hunig, *The storm has cleared: lessons from the CD28 superagonist TGN1412 trial*, 12(5) Nat Rev Immunol 317–8 (2012).

18 J. Kim, et al., *Patient-Customized Oligonucleotide Therapy for a Rare Genetic Disease*, 381(17) N Engl J Med 1644–52 (2019).

19 T. B. Johnson, et al., *Therapeutic landscape for Batten disease: current treatments and future prospects*, 15(3) Nat Rev Neurol 161–78 (2019).

20 J. Kim, et al., *Patient-Customized Oligonucleotide Therapy for a Rare Genetic Disease*, 381(17) N Engl J Med 1644–52 (2019).

21 H. Li, et al., *An oligonucleotide synthesizer based on a microreactor chip and an inkjet printer*, 9(1) Sci Rep 5058 (2019).

22 L. E. Crowell, et al., *On-demand manufacturing of clinical-quality biopharmaceuticals*, Nat Biotechnol (2018).

23 M. H. Aldosari, et al., *Feasibility Study for Bedside Production of Recombinant Human Acid alpha-Glucosidase: Technical and Financial Considerations*, 21(6) Curr Pharm Biotechnol 467–9 (2020).

24 R. T. Schooley, et al., *Development and Use of Personalized Bacteriophage-Based Therapeutic Cocktails To Treat a Patient with a Disseminated Resistant Acinetobacter baumannii Infection. Antimicrobial Agents and Chemotherapy*, (2017).

25 D. M. Pacia, et al., *CRISPR-phage antibacterials to address the antibiotic resistance crisis: scientific, economic, and regulatory considerations*, Journal of Law and the Biosciences (2024).

26 L. Kempf, J. C. Goldsmith, & R. Temple, *Challenges of developing and conducting clinical trials in rare disorders*, Am J Med Genet A (2017).

27 See for example for the USA: US Food & Drug Administration (FDA), Development & Approval Process – Drugs, https://www.fda.gov/drugs/development-approval-process-drugs; for the EU: European Medicines Agency (EMA), Authorization of medicines. Available from: https://www.ema.europa.eu/en/about-us/what-we-do/authorisation-medicines; for Switzerland: Swissmedic, Authorization of human medicines. Available from: https://www.swissmedic.ch/swissmedic/en/home/humanarzneimittel/authorisations.html; for Germany: Federal Institute for Drugs and Medical Devices, Licensing – Evaluating medicinal products. Available from: https://www.bfarm.de/EN/Medicinal-products/Licensing/_node.html.

28 See for example for the USA: US Food & Drug Administration (FDA), The Drug Development Process- Available at: https://www.fda.gov/patients/learn-about-drug-and-device-approvals/drug-development-process; for the EU: European Medicines Agency (EMA), Human regulatory. Available at: https://www.ema.europa.eu/en/human-regulatory/research-development/clinical-trials-human-medicines for Switzerland: Swissmedic, Clinical trails on medicinal products. Available at: https://www.swissmedic.ch/swissmedic/en/home/humanarzneimittel/clinical-trials/clinical-trials-on-medicinal-products.html.

29 For an overview see European Medicines Agency (EMA), Human regulatory. Available at: https://www.ema.europa.eu/en/human-medicines-regulatory-information.

30 Federal Act of 15 December 2000 on Medicinal Products and Medical Devices (Therapeutic Products Act, TPA), SR 812.21. Available at: https://www.fedlex.admin.ch/eli/cc/2001/422/en.

31 For an overview see Swissmedic, The tasks: - Medicinal products and medical devices: from development to market surveillance. Available at: https://www.swissmedic.ch/swissmedic/en/home/about-us/swissmedic--swiss-agency-for-therapeutic-products/patients-and-users.html.

32 Swissmedic, Guidance document: Authorisation of human medicinal product with new active substance, ZL 101_00_005, valid from 1 May 2023. Available from: https://www.swissmedic.ch/swissmedic/en/home/kpa/zulassungsverfahren.html.

33 See for example Paris Valérie & Docteur Elisabeth, Pharmaceutical Pricing and Reimbursement Policies in Switzerland, OECD Health Working Papers No. 27, Paris 2007. Available at: https://www.oecd.org/switzerland/38868953.pdf.

34 See Swissmedic, Revision of the Therapeutic Products Licensing Requirements Ordinance. Available at: https://www.swissmedic.ch/swissmedic/en/home/news/mitteilungen/amzv-nachfuerung-anhang-7.html

35 Swissmedic, Wegleitung Orphan Drug HMV4, Swissmedic, 2021.

36 Verordnung des Schweizerischen Heilmittelinstituts über die vereinfachte Zulassung von Arzneimitteln und die Zulassung von Arzneimitteln im Meldeverfahren (Ordinance of the Swiss Agency for Therapeutic Products on the simplified authorisation of medicinal products and the authorization of medicinal products in the notification procedure), VZAV, SR 812.212.23.

37 Swissmedic, Merkblatt über nichtstandardisierbare Arzneimittel und zur Liste von nichtstandardisierbaren Arzneimitteln und Arzneimittelgruppen mit zulassungspflichtigem Herstellungsverfahren nach Anhang 3 VAZV, Swissmedic, 2020.

38 See Bundesamt für Gesundheit (BAG), Heilversuche. Available at: file:///C:/Users/Claudia/Downloads/Heilversuch_BR-Bericht_DE_webseite-1.pdf.

39 European Commission, A pharmaceutical strategy for Europe of 26 April 2020. Available at: https://health.ec.europa.eu/medicinal-products/pharmaceutical-strategy-europe_en.

40 European Parliament, EU Legislation in Progress, Revision of EU pharmaceutical legislation. Available from: https://www.europarl.europa.eu/RegData/etudes/BRIE/2023/749789/EPRS_BRI(2023)749789_EN.pdf

41 Regulation (EC) No 1394/2007 of the European Parliament and of the Council of 13 November 2007 on advanced therapy medicinal products and amending Directive 2001/83/EC and Regulation (EC) No 726/2004. Available from: https://eur-lex.europa.eu/legal-content/EN/TXT/PDF/?uri=CELEX:32007R1394&qid=1693504424121.

42 European Medicines Agency (EMA), Advanced therapy medicines: exploring solutions to foster Advanced therapy medicines: exploring solutions to foster development and expand patient access in Europe Outcome of a multi-stakeholder meeting with experts and regulators held at EMA on Friday 27 May 2016. Available at: https://www.ema.europa.eu/en/documents/report/outcomes-workshop-multi-stakeholder-advanced-therapy-medicinal-products-atmps-expert-meeting_en.pdf-

43 European Medicines Agency (EMA), EMA Regulatory Science to 2025, Reference Material, 23 October 2018, EMA/723198/2018, Science Committees Regulatory Science Strategy. Available at: https://www.ema.europa.eu/en/documents/other/reference-material-multistakeholder-workshop-launch-consultation-european-medicines-agency-ema-human_en.pdf.

44 EMA Regulatory Science to 2025, p. 5.

45 EMA Regulatory Science to 2025, p. 5.

46 EMA Regulatory Science to 2025, p. 10.

47 EMA Regulatory Science to 2025, p. 10.

48 EMA Regulatory Science to 2025, p. 10.

49 Codex DNA, I. *Welcome to the future* 2021. Available from: https://codexdna.com/products/bioxp-system/.

50 Kilobaser. *Kilobaser Express DNA Machin 2021*. Available from: https://www.kilobaser.com/.

51 K. S. Boles, et al., *Digital-to-biological converter for on-demand production of biologics*, 25(7) Nat Biotechnol 672–5 (2017).

52 P. Perez-Pinera, et al., *Synthetic biology and microbioreactor platforms for programmable production of biologics at the point-of-care*, 7 Nat Commun 12211 (2016).

53 L. E. Crowell, et al., *On-demand manufacturing of clinical-quality biopharmaceuticals*, Nat Biotechnol (2018).

54 H. Schellekens, et al., *Erratum: Making individualized drugs a reality*, 35(8) Nat Biotechnol 797 (2017).

55 H. Maassen, ‘*Medicijnen op maat bereid je magistraal*’, 2017. Available from: https://www.medischcontact.nl/nieuws/laatste-nieuws/artikel/medicijnen-op-maat-bereid-je-magistraal.htm.

56 C. H. Arnaud, *Making biologics on demand*. 2018, Available from: https://cen.acs.org/biological-chemistry/biotechnology/Making-biologics-demand/96/i45.

